# Disturbance of calcium homeostasis and myogenesis caused by TET2 deletion in muscle stem cells

**DOI:** 10.1038/s41420-022-01041-1

**Published:** 2022-04-30

**Authors:** Haoyuan Zhang, Sheng Wang, Qiangwei Zhou, Yinlong Liao, Wenzhe Luo, Zhelun Peng, Ruimin Ren, Heng Wang

**Affiliations:** 1 Key Laboratory of Agricultural Animal Genetics, Breeding and Reproduction of the Ministry of Education, College of Animal Science and Technology, Huazhong Agricultural University, Wuhan, China; 2 Agricultural Bioinformatics Key Laboratory of Hubei Province, Hubei Engineering Technology Research Center of Agricultural Big Data, 3D Genomics Research Center, College of Informatics, Huazhong Agricultural University, Wuhan, China

**Keywords:** Muscle stem cells, Developmental biology

## Abstract

Skeletal muscle myogenesis is a sophisticated process controlled by genetic and epigenetic regulators. In animals, one of the key enzymes for the DNA demethylation of 5-methylcytosine is TET2. Although TET2 is essential for muscle development, the mechanisms by which TET2 regulates myogenesis, particularly the implication for muscle stem cells, remains unclear. In the present study, we employed the TET2 knockout mouse model to investigate the function of TET2 in muscle development and regeneration. We observed that TET2 deficiency caused impaired muscle stem cell proliferation and differentiation, resulting in the reduction in both myofiber number and muscle tissue size. Specifically, TET2 maintains calcium homeostasis in muscle stem cells by controlling the DNA methylation levels of the calcium pathway genes. Forced expression of the sodium/calcium exchanger protein SLC8A3 could rescue the myogenic defects in TET2 knockout cells. Our data not only illustrated the vital function of TET2 during myogenesis but also identified novel targets that contribute to calcium homeostasis for enhancing muscle function.

## Introduction

DNA methylation, the most prevalent epigenetic modification of DNA, plays a major role in regulating gene transcription during the development and regeneration of mammalian tissues [1–3]. The DNA methylation and demethylation are regulated by several enzymes, such as DNA methyltransferase (DNMT) and ten-eleven translocation (TET). The TET family of methylcytosine dioxygenases is mainly involved in the regulation of DNA demethylation processes. It is comprised of three isomeric proteins–TET1, TET2, and TET3–with varying properties in different cells and tissues. The function of these enzymes is to perform iterative oxidation of 5mC to produce 5-hydroxymethylcytosine (5hmC), then 5-formylcytosine (5fC), followed by 5-carboxylcytosine (5caC) and ultimately unmethylated cytosine (C) [4, 5]. This demethylation action regulates various biological processes in the organism. The TET1/2/3 triple knockout mice exhibited premature fetal failure and teratomas due to the dysregulation of genes associated with embryonic development and hypermethylation of their promoter region [6]. Further, TET2/3 double-knockout mice suffered myocardial damage and fetal mortality [7]. The TET1 single knockout caused the dysregulated expression of critical genes in the osteogenic and adipogenic differentiation [8]. It has also been reported that the deletion of TET2 in mouse embryonic stem cells reduced 5hmc levels around pluripotency enhancers and subsequently impaired the activity of these enhancers [9]. In terms of reproductive development, TET1 deficiency causes the reduction in the development of spermatogonia and triggers premature aging of the reproductive system [10]. There have also been reports that TET2 is involved in the process of myogenic differentiation, and its absence was detrimental to muscle development [11].

Skeletal muscle development is a highly sophisticated and complex process involving the epigenetic modification of DNA and chromatin at multiple levels. These, in turn, regulate the specification, proliferation and differentiation of myogenic cells. The DNMT and TET controlled DNA methylation and demethylation play an integral role in the process of myogenesis [12–15]. TET1 and TET2 were highly expressed in muscle cells of different types and species [15]. During skeletal muscle development in pigs, the overall DNA methylation level decreases during the period from embryonic to juvenile and adulthood, possibly due to the downregulation of DNMT1 [3]. Dynamic changes in DNA methylation could affect the expression of myogenic genes by modulating the accessibility of upstream transcription factors (TF) binding sites. For instance, DNA demethylation promotes the expression of myogenic factors (e.g., MyoD), thereby enhancing myogenic capacity [16]. Notably, the transition from quiescence to activation of skeletal muscle satellite cells is usually accompanied by alterations in metabolic status, encompassing changes in the activities of TET and DNMT enzymes [5, 17].

Although previous studies have identified the DNA methylation profile of myogenic cells, the mechanism regarding how specific demethylation-related enzymes such as the TET family genes influence the myogenesis of muscle stem cells has not been investigated in detail. In this study, we demonstrated that TET2 deficiency impaired muscle stem cell proliferation and differentiation, leading to notable changes in mouse body weight and muscle morphology during development. We further assayed the genome-wide DNA methylation profiles associated with TET2 deletion during myogenesis. More specifically, we identified the critical role of TET2 in the regulation of the calcium pathway genes through DNA methylation to control the myogenesis during both development and regeneration

## Result

### TET2 deficiency impairs muscle morphology and myofiber formation in mice

First, TET2-knockout (TET2-KO) mice were produced using the CRISPR/Cas9 technique. In brief, we introduced double-strand breaks into the TET2 locus and the subsequent non-homologous recombination repair process partially deleted exon 3 of the TET2 gene, thus causing the functional inactivation of TET2 (Fig. S1A). PCR and RT-qPCR analysis showed that the TET2 gene was genotypically deficient and minimally expressed in the knockout mice (Fig. S1B). In addition, we examined the expression levels of TET1 and TET3 and found that the expression levels of TET1 and TET3 in the muscle tissue from TET2-KO mice remained constant compared to that of wild type (WT) mice (Fig. S1C). We noticed that the TET2^-/-^ mice in previous studies displayed visible defects in immune tissue and hematopoietic stem cells [18, 19]. Therefore, we also collected thymus and spleen tissue from TET2-KO mice for morphological verification. The results demonstrated that TET2-KO mice exhibited marked splenomegaly, while the thymus of TET2-KO mice showed similar histological features to those of WT mice (Fig. S1D).

**Fig. 1:**
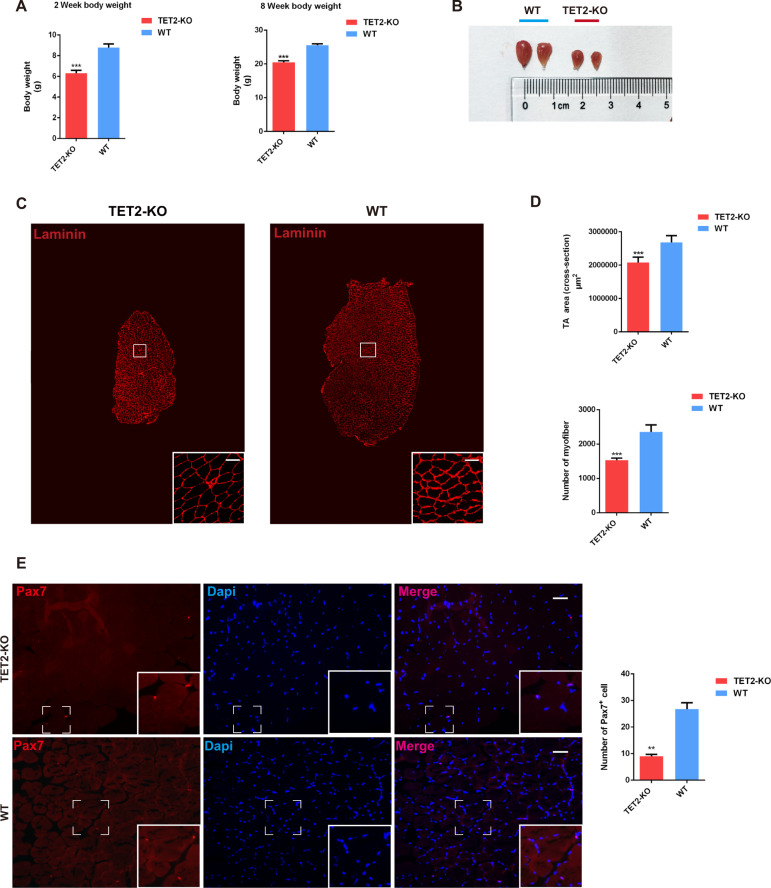
TET2 deficiency affects morphology and muscle development in mice. **A** Quantification of body weight of 2-week and 8-week-old TET2-KO and WT mice, respectively (*n* = 6 mice). **B** Representative images of TA muscles from 8-week-old WT and TET2-KO mice. **C** Immunofluorescence staining of laminin for TA muscles from 8-week-old WT and TET2-KO mice (Scale bars, 50 μm) **D** Quantification of cross-section area and myofiber numbers for TA muscles from 8-week-old WT and TET2-KO mice (*n* = 6 mice). **E** Immunofluorescence staining of PAX7 of TA muscles from 8-week-old WT and TET2 KO mice (Scale bars, 50 μm) and quantification of the number of PAX7^+^ cell per mm^2^ (*n* = 3 biological samples). Error Bar indicated SEM and ** indicated that *p* < 0.01, *** indicated that *p* < 0.001.

To assess whether TET2 deficiency adversely affects muscle development in mice, we comparatively analyzed the muscle phenotype in TET2-KO and WT mice at different developmental stages (Fig. S1E, S1F). Weight measurements indicated a significant reduction in body weight in TET2-KO mice compared with WT mice at 2 and 8 weeks old (Fig. 1A). Interestingly, the tibialis anterior (TA) muscle tissue harvested from 8-week-old mice demonstrated a pronounced reduction in size and morphology in the TET2-KO group (Fig. 1B). The cross-sectional area of the TA muscles in TET2-KO mice was considerably smaller than in WT mice due to the decreased number of myofibers (Fig. 1C, D). In contrast, laminin immunofluorescence staining of TA tissue sections harvested from the two groups of mice revealed that they were comparable in terms of average myofiber cross-sectional area (Fig. S1G, S1H). Given that the cross-sectional area of TA tissues and the number of myofibers for TET2-KO mice were reduced, we then investigated whether TET2 deletion also affected the number of skeletal muscle stem cells (MuSCs) which are associated with and give rise to myofibers. Paired box 7 protein (Pax7) is the canonical transcription factor that specifies and marks the MuSCs [20]. We performed immunostaining of Pax7 for TA tissue, which showed that the number of MuSCs was markedly reduced in TET2-KO mice (Fig. 1E). Taken together, these results show that the deletion of TET2 impairs muscle development and causes myogenic stem cell deficiency.

### TET2 is necessary for muscle stem cell proliferation and muscle development

**Fig. 2:**
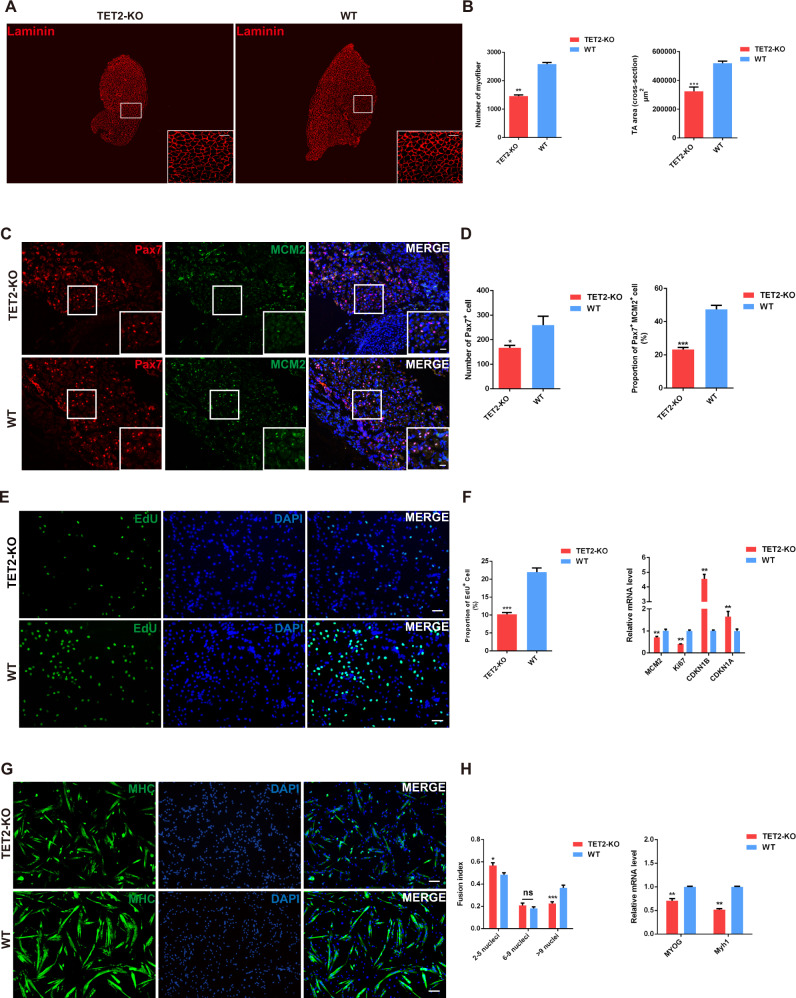
TET2 knockout affects early skeletal muscle development by inhibiting MuSC proliferation and differentiation. **A** Immunofluorescence staining of Laminin of TA muscles from 1-week-old WT and TET2 KO mice (Scale bars, 50 μm) **B** Quantification of the number of myofibers and cross-section area of TA muscles isolated from 1-week-old WT and TET2 KO mice (*n* = 3 biological samples) **C** Immunofluorescence staining of PAX7 and MCM2 of muscle tissue from neonatal WT and TET2 KO mice (Scale bars, 25 μm) **D** Quantification of the number of PAX7^+^ cell per mm^2^ and the percentage of PAX7^+^ MCM2^+^ cell in neonatal WT and TET2 KO mice (*n* = 6 biological samples) **E** Fluorescence staining of EdU in fresh-isolated MuSCs from WT or TET2 KO mice (Scale bars, 50 µm). **F** Quantification of the percentage of EdU^+^ MuSCs (*n* = 3 biological samples) and the relative expression levels of the proliferation related genes in WT and TET2-KO MuSCs. (*n* = 3 biological samples) **G** Immunofluorescence staining of MHC in differentiated myotubes from WT and TET2-KO MuSCs (Scale bars, 50 µm). **H** Quantification of the fusion index in differentiated myotubes (*n* = 3 biological samples) and relative expression levels of the myogenic differentiation related genes in WT and TET2-KO MuSCs (*n* = 3 biological samples). Error Bar indicated SEM. ns indicated that Not Significant, * indicated that *p* < 0.05, ** indicated that *p* < 0.01, *** indicated that *p* < 0.001.

Following the observation regarding the phenotypic differences in muscle tissue between the adult TET2-KO and WT mice, we next determined when this discrepancy occurred during early muscle development. First, we harvested TA tissues from 1-week-old mice for further analysis. In terms of morphology, the cross-sectional area of TA tissue obtained from 1-week-old TET2-KO mice was proportionately smaller than that of WT mice, and the number of myofibers in the TET2-KO group was also considerably reduced (Fig. 2A, B).

To investigate whether TET2 elimination also causes changes in MuSCs in the early stages of muscle development, we examined the Pax7^+^ cells in the TA muscle of 1-week-old mice. MCM2 is a component of the minichromosome maintenance (MCM) complex, which is essential for the initiation and elongation of DNA and commonly employed to mark the proliferating cells [21]. Both the number of Pax7^+^ MuSCs and the proportion of Pax7^+^ MCM2^+^ double-positive proliferating MuSCs were dramatically reduced in TET2-KO mice (Fig. S2A, S2B, S2C). Further, we collected leg muscles from newborn mice to investigate the profile of MuSCs. Correspondingly, we also observed a remarkable decrease in the number of PaX7^+^ MuSCs and their proliferation rate in TET2-KO mice (Fig. 2C, D). Taken together, these results demonstrate that TET2 deficiency impedes muscle stem cell generation and proliferation during early muscle development.

To directly investigate the effect of TET2 deletion on MuSCs, we examined the proliferation and differentiation capacity of the freshly-isolated MuSCs from both TET2-KO and WT mice. The expression levels of TET1 and TET3 in isolated TET2-KO MuSC were constant, indicating that other TET members could not compensate for the TET2 loss in muscle (Fig. S2D). We identified homogenous Pax7 expression in isolated MuSCs and confirmed the purity of cells isolated from both types of mice (Fig. S2E). Subsequently, 5-Ethynyl-2’- deoxyuridine (EdU) staining revealed a remarkable reduction in the percentage of EdU^+^ cells upon TET2 deletion (Fig. 2E, F). Previous studies have shown that Ki67 protein has been widely used as a marker of cell proliferation while CDKN1B and CDKN1A were the main negative regulators for the cell cycle [22, 23]. RT-qPCR results demonstrated that the expression of proliferation markers such as *MCM2* and *Ki67* were dramatically downregulated while the negative regulators of cell proliferation such as *CDKN1B* and *CDKN1A* were upregulated (Fig. 2F). Given that MyoG is necessary for myogenic cell differentiation and MYH family proteins are predominantly expressed in mature muscle fibers of adult mice, we examined the differentiation capacity of TET2-KO and WT MuSCs by analyzing the expression levels of MyoG and Myh [24, 25]. Upon low-serum differentiation induction, the TET2-KO cells displayed lower myogenic capacity, as shown by the fewer MHC^+^ myotubes (Fig. 2G) and reduced expression of myogenic differentiation-related marker genes such as *MyoG* and *Myh1* (Fig. 2H). Therefore, the TET2 is necessary for both the proliferation and differentiation of MuSCs.

Since Tet2 deficiency impaired the proliferation and differentiation of muscle stem cells, we wondered whether the muscle lineage determination and myogenic specification from the multipotent progenitor cells were also compromised by TET2 deletion. We utilized the mesenchymal stem cells (C3H10T1/2) to evaluate the role of TET2 in the determination of myogenic cell fate and the repression of non-muscle lineages. We used siRNA to knock down the TET2 expression in C3H10T1/2 cells (Fig. S3A). Subsequently, we examined the expression levels of the genes implicated in early osteogenesis (*Sfrp2, Fbn2*) [26, 27], chondrogenesis (*Sox9*) [28] and adipogenesis (*PGC-1α, C/EBPα*) [29]. The qPCR results showed that the adipogenesis genes PGC-1α, C/EBPα were significantly downregulated and the chondrogenesis gene SOX9 expression was not affected. However, the expression of the osteogenesis genes Fbn2 and Sfp2 was dramatically upregulated (Fig. S3B).

To further validate the adipogenic potential upon TET2 inhibition, we induced adipogenic differentiation in siTET2 and control cells. The Oil Red O staining showed that lipid droplet deposition of siTET2 group was considerably lower than that of the control group after lipogenesis induction (Fig. S3C, S3D). The expression levels of the lipogenic differentiation marker genes (*Adipoq,Fabp4,PPARγ*) were also significantly downregulated in the siTET2 group (Fig. S3G). In addition, previous research demonstrated that TET2 could inhibit the osteogenic differentiation of bone marrow mesenchymal stem cells [30]. Similarly, we found that TET2 deficiency facilitated the osteogenic differentiation of C3H10T1/2 cells (Fig. S3E, S3F). The qPCR result also showed that the expression level of osteogenic differentiation marker genes (*Sp7,Runx2,Sfrp2*) was significantly increased upon TET2 inhibition (Fig. S3H). These results indicate that TET2 plays an important role in cell lineage determination of multipotent cells and TET2 dysregulation could affect stem cell fate.

### TET2 knockout mice exhibit delayed muscle regeneration

**Fig. 3:**
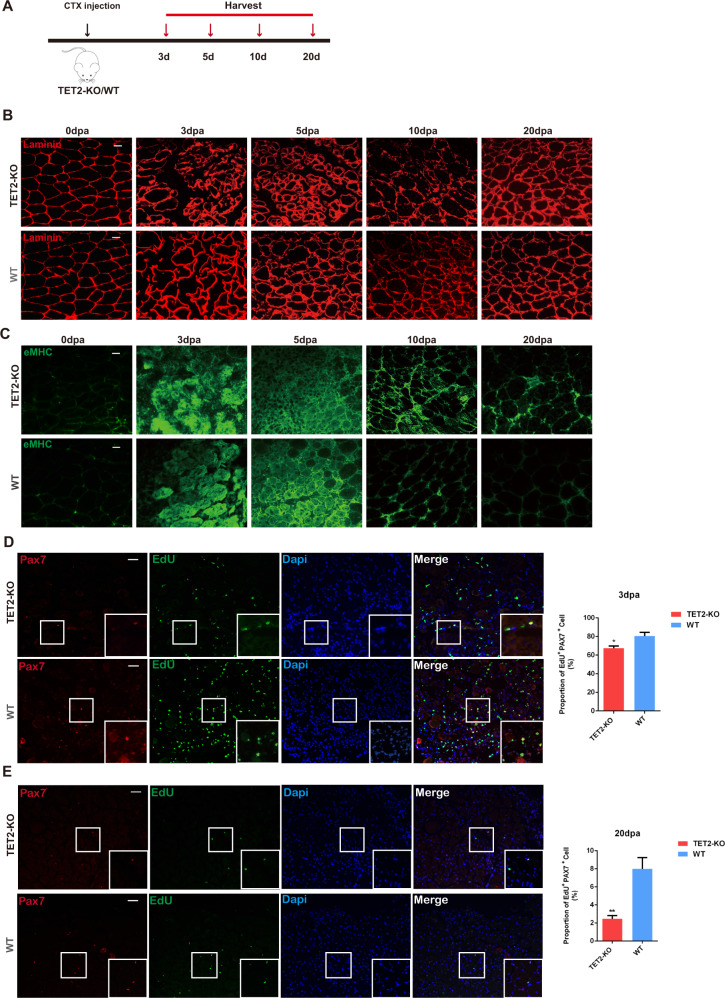
TET2 knockout mice exhibit delayed muscle repair and regeneration due to the impaired MuSC proliferation. **A** Schematic diagram of the muscle regeneration experiment. 100 μl 10 μM CTX was injected into TA tissue of 8-week-old WT and TET2-KO mice. **B** Immunofluorescence staining of laminin of TA muscles after injury (Scale bars, 50 μm). **C** Immunofluorescence staining of eMHC of TA muscles after injury (Scale bars, 50 μm) **D** Immunofluorescence staining of PAX7 and EdU at 3 dpa (Scale bars, 50 μm) and quantification of the percentage of PAX7^+^ EdU^+^ cell (*n* = 3 biological samples). **E** Immunofluorescence staining of PAX7 and EdU at 20 dpa (Scale bars, 50 μm) and quantification of the percentage of PAX7^+^ EdU^+^ cell (*n* = 3 biological samples). Error Bar indicated SEM. * indicated that *p* < 0.05, ** indicated that *p* < 0.01.

Due to the negative effects of TET2 deletion associated with myogenesis both in vivo and in vitro, we next determined whether TET2-deficient mice would also exhibit defects in muscle repair and regeneration. We administered cardiotoxin (CTX) into the adult TA muscle and collected the tissue at 3, 5, 10, and 20 days post-injury (dpi, Fig. 3A). The regeneration defects of TET2-KO mice were observed as indicated by laminin staining (Fig. 3B). Concurrently, the embryonic MHC (eMHC) staining also indicated retardation of the muscle repair in TET2-KO mice (Fig. 3C). Next, we performed double-staining of Pax7 and EdU at different time points after injury to investigate the activation and proliferation dynamics of MuSCs. The results revealed that TET2 deletion caused a considerable decrease in the activation and proliferation rates of MuSCs at 3- and 20-day post-injury (Fig. 3D, E). Therefore, we concluded that TET2 was also necessary for muscle regeneration, particularly for the activation and proliferation of MuSCs.

### Transcriptome analysis of MuSCs with TET2 deficiency

**Fig. 4:**
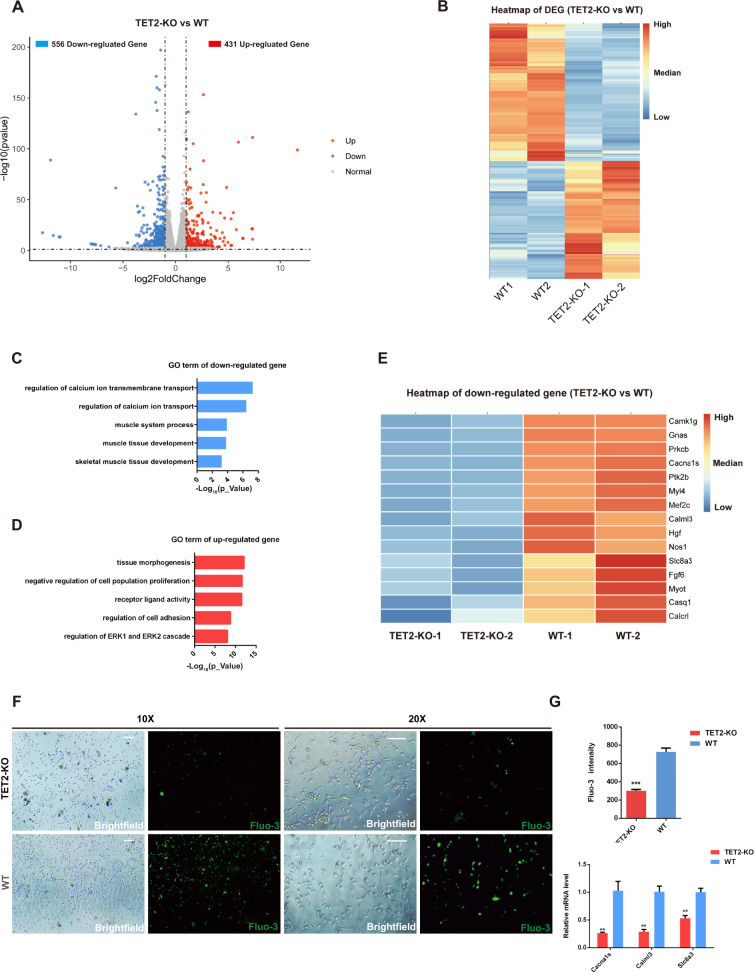
Transcriptome analysis of MuSCs upon TET2 loss. **A** The volcano plot of DEGs (TET2-KO versus WT). **B** Heatmap of the expression level of DEG. **C** Gene ontology analysis of downregulated gene in TET2 KO group. **D** Gene ontology analysis of upregulated gene in TET2 KO group. **E** Heatmap of the most down-regulated genes. **F** The fluorescence labeling of Fluo-3 probe in the MuSCs of TET2-KO and WT group (Scale bars, 100 μm). **G** Quantification of the intensity of Fluo-3 (*n* = 6 biological samples) and relative expression levels of calcium-signaling related genes in WT and TET2-KO MuSCs (*n* = 3 biological samples). Error Bar indicated SEM. ** indicated that *p* < 0.01, *** indicated that *p* < 0.001.

To further uncover the underlying mechanisms of TET2-KO defects that affect myogenesis, we performed RNA-seq on isolated TET2-KO and WT MuSCs to identify the gene expression alterations associated with TET2 deletion. Principal component analysis (PCA) demonstrated that the overall transcriptome of the TET2-KO group was fundamentally different from that of the WT group (Fig. S4A). We identified 987 differentially expressed genes (DEGs), of which 431 were upregulated and 556 were downregulated upon TET2 knockout (Fig. 4A, B). Meanwhile, gene ontology (GO) analysis of the DEGs revealed that the upregulated genes were predominantly enriched in biological functions such as tissue morphogenesis and negative regulation of cell proliferation, while the downregulated genes were mainly enriched in muscle tissue development and regulation of calcium ion transmembrane transport (Fig. 4C, D). Interestingly, both GO and KEGG analyses indicated that, in addition to the myogenic genes, the calcium pathway genes such as *Slc8a3, Cacna1s* were most downregulated. (Figs. 4E, S4B). Given that the calcium signaling pathway-related genes displayed remarkable dysregulation due to TET2 ablation (Fig S4C), we utilized the cell-permeant Fluo-3 AM Ca^2+^ indicator probe to measure the intracellular calcium concentration in TET2-KO and WT cells. We discovered that the calcium level in TET2-KO MuSCs was considerably lower than in WT MuSCs. Quantitative PCR also confirmed that expression levels of calcium pathway-related genes were drastically reduced upon TET2 deletion (Fig. 4F, G). These results indicated that TET2 deletion caused drastic alterations in gene expression and disrupted the calcium homeostasis in MuSCs.

### Calcium signaling pathway-related genes are hypermethylated in TET2 deficient MuSCs

**Fig. 5:**
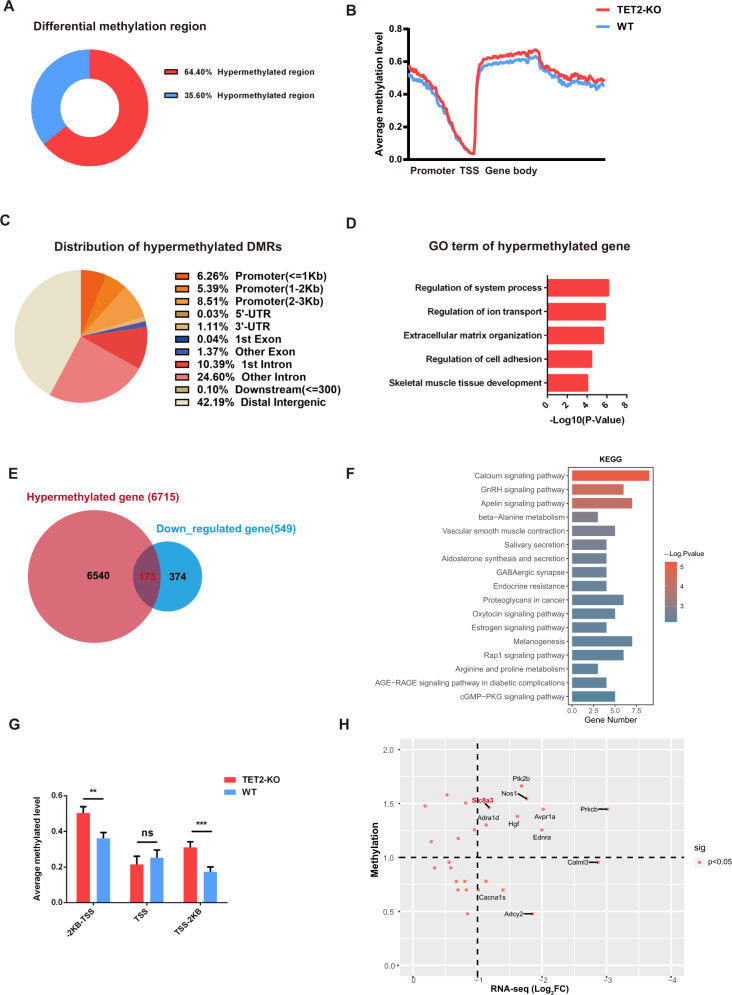
Calcium signaling pathway related genes are hypermethylated in TET2 deficient MuSCs. **A** Proportion profile of the differentially methylated regions (DMRs) (TET2-KO versus WT) **B** Average methylation level of gene region. **C** Distribution of the DMRs at various genomic regions. **D** Gene ontology analysis of hypermethylated gene in TET2 KO group. **E** The venn diagram of hypermethylated and down-regulated genes in TET2 KO group. **F** KEGG analysis of the 175 hypermethylated and downregulated genes in TET2 KO group. **G** Average methylated level across the TSS of calcium signaling genes. **H** Scatter plot of expression and methylation levels of the calcium-signaling genes in TET2 KO group. Error bar indicated SEM. ns indicated that not significant; ** indicated that *p* < 0.01, *** indicated that *p* < 0.001.

Since the TET protein family is mainly involved in DNA demethylation, we performed whole-genome methylation sequencing (WGBS) on isolated MuSCs to reveal the methylation profiles in TET2-KO cells. We identified 11,502 differentially methylated regions (DMR) upon TET2 deletion, of which 64.4% were hypermethylated (Fig. 5A). Interestingly, the methylation levels around the coding genes were slightly elevated upon TET2 deletion, particularly in the promoter region (Fig. 5B). However, most of the hypermethylated DMRs were located in introns and distal intergenic regions (Fig. 5C). GO analysis of the hypermethylated genes suggest that they are linked to the regulation of ion transport, cell adhesion, and skeletal muscle tissue development (Fig. 5D). Furthermore, integrated analysis between the hypermethylated gene loci and downregulated genes identified 175 candidate genes that could be directly targeted by TET2 demethylation since TET2 deletion leads to higher methylation and transcriptional silencing of these genes (Fig. 5E). Further KEGG analysis revealed that these genes were predominantly enriched in the calcium pathway (Fig. 5F). Conversely, the KEGG results of the repressed genes without overlap with hypermethylated genes were not enriched explicitly in any signaling pathways (data not shown). To further validate the aberrant DNA methylation modifications that may affect the calcium pathway genes, we analyzed the methylation levels of the region ±2 KB around the transcriptional start sites (TSS) of representative calcium pathway genes. We observed higher methylation levels in the promoter and gene body regions in TET2-KO cells (Fig. 5G). Subsequently, we carried out a correlation analysis between the mRNA expression levels and the methylation status of the calcium pathway genes to identify functional candidates that could play a significant role in TET2-mediated calcium transportation and muscle development. Our results showed that *Prkcb, Slc8a3, Avpr1a*, and *Ednra* stood out among the most hypermethylated and downregulated genes (Fig. 5H). During the maintenance of integral homeostatic calcium regulation, Slc8a3 was considered a major candidate factor for causing calcium signaling pathway defects in TET2-KO MuSCs. These data suggest that TET2 was necessary for maintaining the normal MuSC function, particularly through the DNA methylation regulation of genes in the calcium pathway.

### Slc8a3 partially rescues the myogenic capacity of TET2-KO MuSCs

Slc8a3 is regarded as an integral protein in maintaining the intracellular calcium homeostasis [31] since it acts as a potent regulator of Ca^2+^ transfer [32–34]. Specifically, the Slc8a3 protein regulates the entry of calcium ions into myocytes through an ion-exchange mechanism [34, 35]. Considering that TET2-KO and WT MuSCs displayed dramatic differences in calcium homeostasis, we explored whether the impaired myogenic capacity in TET2-KO MuSCs can be restored by forcibly elevating the expression of calcium pathway genes. The profile of calcium ion concentration in MuSCs (Fig. 4F, H) and the DNA methylation status of Slc8a3 was most impacted in our methylation data (Fig. 5H). Therefore, it is reasonable to assume that *Slc8a3* is the primary candidate gene for improving the calcium homeostasis in TET2-KO MuSCs.

KB-R7943, a widely used inhibitor of Slc8a3, interferes with sodium/calcium exchanger protein mainly by interacting with domains other than the ion transport site [36]. We treated WT MuSCs with KB-R7943 to verify whether Slc8a3 deficiency indeed impaired the calcium homeostasis and myogenic functions in MuSCs. The results of Fluo-3 AM fluorescence labeling illustrated that the intracellular calcium ion concentration of WT MuSCs was considerably diminished after KB-R7943 treatment (Fig. S5A, S5B). In addition, RT-qPCR of the selective genes involved in calcium signalings indicated that their expression levels were down-regulated. (Fig. S5E). Further, EdU staining showed impaired proliferation of MuSCs after application of KB-R7943 (Fig. S5C, S5D), which is consistent with the qPCR results of the expression of cell proliferation-associated transcription factors (Fig. S5E). We also discovered that the KB-R7943 treatment exerted negative regulation on the expression of myogenic transcription factors (Fig. S5E). Altogether, this Slc8a3 inhibition assay demonstrated that Slc8a3 is required for the proliferation and differentiation capacity of MuSCs by maintaining calcium homeostasis.

**Fig. 6:**
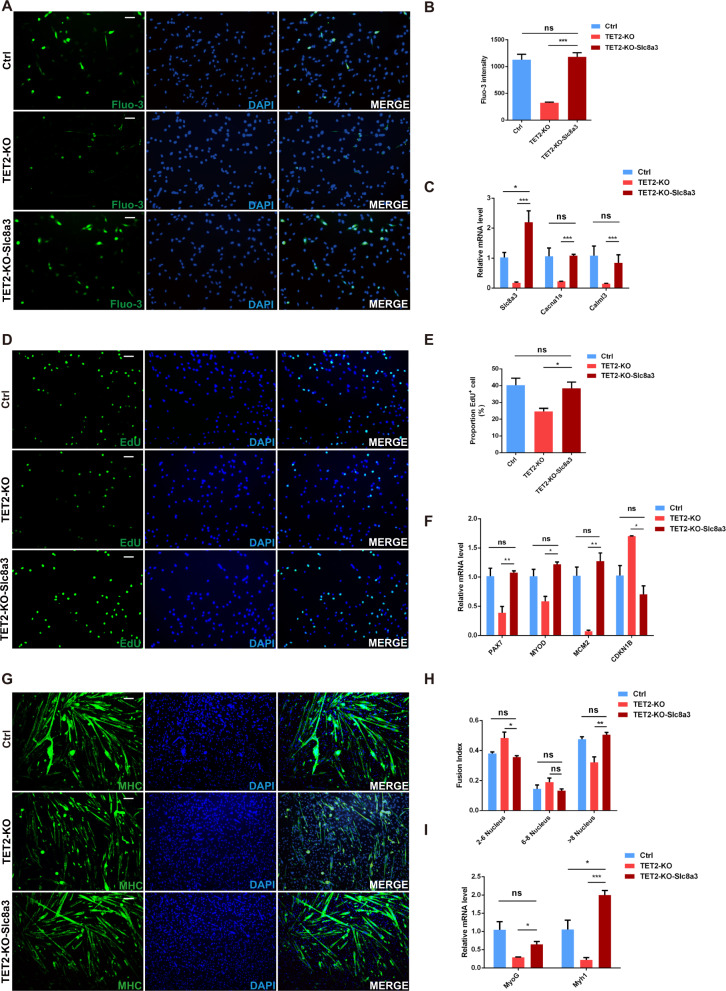
Slc8a3 partially rescues the myogenic capacity of TET2-KO MuSCs. **A** The Fluo-3 probe of Ctrl, TET2-KO and TET2-KO-Slc8a3 group (Scale bars, 50 µm). **B** Quantification of the intensity of Fluo-3 of Ctrl, TET2-KO, and TET2-KO-Slc8a3 group (*n* = 3 biological samples). **C** Relative expression levels of Ca-signaling related genes in Ctrl, TET2-KO and TET2-KO-Slc8a3 group (*n* = 3 biological samples). **D** Immunofluorescence staining of EdU in Ctrl, TET2-KO, and TET2-KO-Slc8a3 group (Scale bars, 50 µm). **E** Quantification of the percentage of EdU^+^ in Ctrl, TET2-KO, and TET2-KO-Slc8a3 group (*n* = 3 biological samples). **F** Relative expression levels of the proliferation related genes in Ctrl, TET2-KO, and TET2-KO-Slc8a3 group (*n* = 3 biological samples). **G** Immunofluorescence staining of MHC in differentiated myotubes from Ctrl, TET2-KO, and TET2-KO-Slc8a3 group (*n* = 3 biological samples). **H** Quantification on the fusion index of differentiated myotubes from Ctrl, TET2-KO, and TET2-KO-Slc8a3 group (*n* = 3 biological samples). **I** Relative expression levels of the myogenic related genes in Ctrl, TET2-KO, and TET2-KO-Slc8a3 group (*n* = 3 biological samples). Error Bar indicated sem ns indicated that Not Significant, * indicated that *p* < 0.05, ** indicated that *p* < 0.01, *** indicated that *p* < 0.001.

Based on the above Slc8a3 loss-of-function assays, we hypothesized that Slc8a3 could serve as a potential effector gene in improving the calcium regulation and myogenic capacity of TET2-KO MuSCs. Next, we investigated whether the impaired TET2-KO MuSCs can be revived through the exogenous overexpression of Slc8a3. We transfected freshly-isolated TET2-KO and WT MuSCs with Slc8a3-overexpression and control plasmids, respectively, and then assessed the calcium concentration, cell proliferation, and myogenic capacity (Fig. S6A). The qPCR results showed that the Slc8a3 expression level was significantly upregulated after the gene transfection in TET2-KO MuSCs (Fig. S6B). In terms of calcium regulatory capacity, we discovered that the intracellular Ca^2+^ concentration of TET2-KO-Slc8a3 MuSCs was dramatically increased compared to TET2-KO MuSCs (Fig. 6A, B). Correspondingly, the expression levels of calcium signaling-related genes, such as *Slc8a3, Cacna1s* and *Calml3*, displayed a notable elevation (Fig. 6C). EdU staining also indicated that the cell proliferation rate of TET2-KO cells was substantially elevated after Slc8a3 overexpression, reaching levels comparable to the WT (Fig. 6D, E). Accordingly, the cell proliferation was recovered in the Slc8a3 overexpressing cells as shown by the elevated expression of *MCM2* gene and the downregulation of the cyclin-dependent kinase inhibitor gene *CDKN1B*. In addition, the expression levels of myogenic transcription factors were also elevated in the TET2-KO-Slc8a3 group (Fig. 6F). Moreover, in vitro differentiation experiments revealed that Slc8a3 overexpression could rescue the myogenic differentiation defects of TET2-KO cells as shown by the similar fusion index between the WT and TET2-KO-Slc8a3 groups (Fig. 6G, H). Accordingly, the expression level of typical myogenic differentiation-associated genes also showed remarkable elevation in Slc8a3-overexpressing cells (Fig. 6I). Altogether, these results suggested that exogenous Slc8a3 expression rescued the calcium homeostasis defects in TET2-KO MuSCs and consequently restored the cell proliferation and myogenic properties. It also demonstrated the critical role of Slc8a3 in the calcium regulation and myogenic capacity of MuSCs.

## Discussion

Previous studies have shown that TET family enzymes play important roles in embryonic stem cells, spermatogonial stem cells, hematopoietic stem cells, and cardiac progenitor cells. Thus, the TET family of proteins affects the early development of different tissues and organs [7, 10, 37]. Specifically, TET2 primarily regulates 5hmc levels in the gene body, exon boundaries and exons of highly expressed genes in mouse embryonic stem cells [6]. TET deficiency impairs embryonic stem cell differentiation and consequently generates poorly-differentiated germinal bodies and teratomas [38]. Preliminary data also demonstrated that TET2 deficiency impaired the differentiation capacity of myogenic cells [11, 39, 40]. However, a recent study showed that TET2 was only necessary for skeletal muscle regeneration but is dispensable for muscle development [41]. Therefore, in this study, we sought to analyze the muscle tissue and cells during the early developmental stage in TET2 knockout mice to investigate the factors that could be regulated by TET2 in early myogenesis.

Initially, we found that adult TET2-KO mice show signs of abnormal myogenesis. Specifically, the TET2-KO mice showed a reduction in the number of MuSCs, which was caused by the impairment of the proliferative capacity of MuSCs in vivo. The cultured TET2-KO MuSC also showed defects in proliferation and myogenic differentiation. Interestingly, the TET2 inhibition in multipotent mesenchymal stem cells revealed that TET2 dysregulation disrupted the cell stemness and accelerated their osteogenic differentiation, implying that TET2 deletion could diminish the muscle cell lineage through the regulation of cell fate determination other than muscle. We further validated the role of TET2 in muscle injury models and the retardation of muscle regeneration in TET2-KO mice was also in accordance with the previous studies [11, 41]. Moreover, the muscle regeneration process can be affected by various types of cells residing within the tissue, such as fibroblasts and immune cells. Previous studies revealed that TET2 absence caused dysfunction in immune cells [42, 43]. We can’t rule out the possibility that defects in myogenic stem cells and other types of cells both contributed to the deterioration of muscle repair. Nevertheless, considering the critical role of muscle stem cells in muscle regeneration and the cell-autonomous impairment of TET2-KO muscle stem cells both in vivo and in vitro, we could conclude that the TET2-depletion caused malfunction of MuSCs was responsible for the abnormalities in muscle development and regeneration. More importantly, we suspect that the defects in muscle development and regeneration were primarily due to a dysregulation of the expression of myogenic genes caused by aberrant DNA methylation in regulatory regions such as promoter and enhancer. Therefore, we further carried out transcriptomic and genome-wide methylation analysis to unravel the key regulatory regions impacted by DNA methylation.

Previous research has shown that the loss of the TET enzymes causes an aberrant elevation of DNA methylation across multiple regions of the genome, including promoters, enhancers, and other cis-regulatory elements [9, 44, 45]. Our transcriptomic analysis also revealed that most DEGs resulting from the loss of TET2 were downregulated, which is in accordance with the genome-wide methylation results. In view of the critical role of TET2 in demethylation processes [46], we consider that this is possibly because TET2 deletion induces an overall elevation in genomic methylation levels. In addition, GO analyses showed that muscle function-related genes were enriched in the downregulated genes, consistent with the phenotypes of muscle development and regeneration experiments. Notably, the downregulated genes associated with hypermethylated loci were evidently enriched in the calcium signaling pathway. Concurrently, the results of the calcium probe indicator assays confirmed that calcium homeostasis was indeed disrupted in TET2-KO MuSCs. Therefore, we reasoned that calcium signaling pathway-associated genes were primarily affected by the abnormal DNA methylation and are thus being repressed in TET2-KO MuSCs.

Calcium signaling is extensively involved in muscle stem cell maintenance as well as the muscle development and regeneration [47–50]. For instance, Hara M et al. demonstrated that calcium influx stimulates satellite cell activation through cell membrane ion channels and mechanosensitive cation channels [51]. Furthermore, Liu et al. found that increased calcium influx and cytosolic calcium increased the number of MyoD positive satellite cells per muscle fiber [52]. We subsequently identified several calcium signaling pathway-related genes, such as *Slc8a3, Nos1* and *Prkcb*, that were dramatically downregulated. Moreover, the DNA methylation levels around these genes were elevated in the absence of TET2. Therefore, we reason that TET2-deletion caused DNA hypermethylation and reduced the expression of genes responsible for maintaining calcium homeostasis in MuSCs. Several reports also indicated that these calcium associated genes were intrinsically involved in myogenesis [34, 53–55]. Specifically, Slc8a3 was essential for the maintenance of intracellular calcium homeostasis in the skeletal muscles [34]. The *Slc8a3* gene encodes the Na^+^/Ca^2+^-Exchange Protein 3 (NCX3) protein, a member of the sodium/calcium exchanger integral membrane protein family. NCX3 is mainly located in the plasma membrane and intracellular organelle membranes and maintains intracellular calcium homeostasis in various cells [56–58]. Furthermore, the Slc8a3 displayed a tissue-specific distribution pattern in vivo and in muscle tissue, its expression fluctuates at different developmental stages [58, 59]. Previous studies also showed that the intranuclear localization of Slc8a3 is associated with the cell cycle and that the inhibition of NCX proteins impairs the proliferation of myofibroblasts [60–62]. According to a knockout mouse model, Slc8a3 deletion caused dysregulation of muscle calcium homeostasis and myofiber necrosis [34]. Our transcriptome and DNA methylation data indicated that the mRNA expression of *Slc8a3* was significantly decreased while the methylation level of its gene locus was substantially increased after TET2 knockout. These data demonstrate that TET2 could directly regulate Slc8a3 expression through changes in DNA methylation levels. Further, KB-R7943, the Slc8a3 inhibitor, [63] was used to evaluate the role of Slc8a3 in calcium homeostasis and the myogenic function in MuSCs. We concluded from the loss-of-function experiments that Slc8a3 is necessary for calcium homeostasis as well as the proliferation and differentiation capacity of MuSCs.

We then performed Slc8a3 overexpression experiments to verify whether Slc8a3 can rescue the myogenic defects of MuSCs caused by TET2 deletion. We first confirmed that Slc8a3 could indeed stimulate the activity of the calcium signaling pathway and contribute to the restoration of intracellular calcium concentration. Next, considering the results of previous studies on calcium pathways and muscle development [64, 65] and our Slc8a3 loss-of-function assays, we attempted to restore intracellular calcium homeostasis through Slc8a3 expression in TET2-KO MuSCs as a means of rescuing their myogenic deficiency. We demonstrated that Slc8a3 could efficiently restore the myogenic ability of MuSCs, as shown by the results of the cell proliferation and differentiation assays. Consistently, we observed that the mRNA levels of proliferation- and myogenic differentiation-related genes were elevated upon Slc8a3 overexpression.

Taken together, we concluded that TET2 deletion in MuSCs caused dynamic changes in DNA methylation modifications and the expression of numerous genes, particularly those involved in the calcium pathway. We successfully recovered calcium signaling defects in TET2-KO MuSCs by introducing exogenous Slc8a3, which partially improved both cell growth and myogenic performance. The current study provides a framework for identifying potential targets for improving myogenesis in muscle cells with aberrant epigenetic modifications.

## Materials and methods

### Animal and tissue

All mice were housed in a pathogen-free environment at 24–26 ^◦^C and under a 12 h/12 h light/dark cycle. C57BL/6 were obtained from Laboratory Animal Center of Huazhong Agricultural University. TET2-KO was generated by Shanghai Biomodel Organism Co., Ltd., Shanghai, China. Mice were killed by cervical dislocation, and their TA were harvested for further experiments.

### Immunofluorescence staining

TA muscle samples were embedded in OCT (Thermo Fisher Scientific, 6506), and frozen in liquid nitrogen for 20 s then cut for 5–8 μm thick cryosections. Cryosections or cells were fixed in 4% paraformaldehyde for 5 min at room temperature and washed two times with PBS. Samples were permeabilized with 0.5% Triton X-100 for 10 min and blocked in 5% goat serum (Gibico, 16210072) for 60 min at room temperature. The samples were incubated in primary antibody (Table S1) overnight followed by two-times wash with PBS. This was followed by incubation with the Alexa Fluor-labeled secondary antibody at 1:500 dilution (Table S1) at room temperature for 2 h. Nuclear staining was carried out with 50 μg/mL DAPI for 10 min at room temperature. Fluorescent images were collected using the fluorescence microscope. Pax7 immunofluorescent staining was carried out as described previously [66]. Antigen retrieval was performed using a pressure cooker for 10 min.

### DNA isolation and genotyping

Genotyping of TET2-KO mice was performed by PCR using genomic DNA extracted from muscle tissue by DNA extraction kits (TIANGEN, Beijing) according to the manufacturer’s recommendations. PCR products were amplified by genotyping primer (Table S2) and separated on 2% Tris-acetate agarose gels.

### MuSCs Cell isolation, culture and differentiation

MuSCs were isolated as previously described [67]. Briefly, dissected muscle tissue was digested with 10 ml digestion buffer (DMEM containing 0.125 mg/mL Collagenase II (Gibco, 17101015), and 10 mg/ml Collagenase A (Roch, 10103586001)) in Water Baths Shaker for 60 min at 37 °C. The digestion was stopped by adding 10 mL of DMEM. The digested cells were filtered through 40 μm strainers. The mononuclear cells were cultured on the plastic dish for 30 min and then transferred to corning matrigel (Corning, 356234) coated dish. The MuSCs cells were cultured at 37 °C in 5% CO_2_ in RPMI-1640 medium (Life, A10491) with 20% fetal bovine serum (Gibico, 10270-106), as described previously [67]. For the cell differentiation experiments, the cells were incubated in differentiation medium (DMEM containing 2% horse serum) for 72 h.

### Measurement of myofiber size and fusion index

The myofiber cross-section area was measured by ImageJ software. The number of myofibers and the fusion index (the number of nuclei in differentiated myotubes) were counted by ImageJ software and Graphpad prism 6. The entire TA tissue cross-sectional area was calculated ImageJ software and at least 3 biological samples were taken.

### EdU labeling

MuSCs were incubated with 10 μM EdU for 1 h at 37 °C followed by fixation with 4% paraformaldehyde, permeabilized with 0.5% Triton X-100 for 10 min, then incubated with reaction buffer (100 mM Tris (pH 8.5), 1 mM CuSO4, 100 μM fluorescent azide) for 30 min. For in vivo experiment, 50 μg/g body weight EdU (Invitrogen, E10187) was injected intraperitoneally one day before sacrifice. EdU staining was performed as previously described [68].

### Lipogenic induction and osteogenic induction

C3H10T1/2 cells (Stem Cell Bank, Chinese Academy of Sciences) were cultured at 37˚C with 5% CO_2_ in DMEM (Gibco, Thermo Fisher Scientific) supplemented with 10% FBS (Gibco, Thermo Fisher Scientific) The culture medium was changed every 2 days and the cells were passaged at ~85% confluence. At ~85% confluence, osteogenic differentiation was induced by osteogenic medium (DMEM supplemented with 10% FBS, 10 nM dexamethasone, 10 mM β-glycerophosphate and 50 µg/ml ascorbic acid (Beyotime)) for 7 days at 37˚C with 5% CO_2_. Lipogenic differentiation was induced by lipogenic medium (DMEM supplemented with 10% FBS, 0.5 mM isobutylmethylxanthine (Merck, USA), 125 nM indomethacin, 1 μM dexamethosone, 20 nM insulin, and 1 nM Liothyronine (MCE, USA)). After 2 days, cells were switched to maintenance medium (DMEM supplemented with 10% FBS, 1 nM Liothyronine, 20 nM insulin, 0.5 μM rosiglitazone (MCE, USA)) for 5 d at 37 ˚C with 5% CO_2_.

### Oil Red O staining

Lipid accumulation in adipocytes was observed by Oil Red O staining. Cells were washed three times with PBS, followed by fixation with 4% Paraformaldehyde for 20 min at room temperature. After fixation, the cells were washed three times with PBS and stained with a filtered Oil red O solution (0.5 g Oil Red O (Sigma, USA) in 100 ml isopropyl alcohol) for 30 min at room temperature. The cells were then washed twice with distilled water for 15 min.

### Alizarin red staining

Osteoblast mineralized staining kit (Beyotime, China) was used for alizarin red staining. After induction of cell differentiation, remove the culture medium and wash two times with PBS. The cells were fixed with fixation solution for 20 min and then washed 3 times with PBS. Alizarin red staining solution was used for 30 min at room temperature. The sample was washed well with distilled water, then observed and photographed under the microscope.

### Cardiotoxin (CTX) injection

Muscle injury was induced by intramuscular injections of CTX (MCE, HY-P1902A) into TA muscle as previously described [69]. Each mouse was injected with 100 μL 10 μM CTX at multiple injection sites in TA muscle. Mice were put under suction anesthesia with isoflurane during injection.

### mRNA extraction, real-time quantitative PCR and RNAseq library construction

Total RNA was extracted by QIAsymphony RNA Kit (Qiagen, 931636). RNA concentration was measured on NanoDrop 2000 (Thermo Scientific, USA). 1 μg of RNA was reverse transcribed using PrimeScript™ RT reagent Kit (TAKARA, RR047A). Real-time quantitative PCR (RT-qPCR) was performed using SYBR Green Mix (Abclonal, RK21203) following the manufacturer’s instructions. Expression was normalized to GAPDH using 2^-ΔΔCt^ method. The RT-qPCR primers are designed by Primer-BLAST (https://www.ncbi.nlm.nih.gov/tools/primer-blast/index.cgi) and described in Table S2. The RNAseq library-preparation protocol was based on the NEBNext Ultra™ RNA Library Prep Kit for Illumina (NEB, E7530L). Insert size was assessed using the Agilent Bioanalyzer 2100 system and qualified insert size was accurate quantification using StepOnePlus™ Real-Time PCR System (Library valid concentration > 10 nM) and then paired-end sequencing using an Illumina platform. RNA-Seq was performed twice for each sample and the number of sequences obtained in each replicate ranged from 40-50 million.

### siRNA knockdown

TET2-siRNA sequences were designed according to the mRNA sequence (GenBank XM 006501283.5) with the online design tools (http://sidirect2.rnai.jp/design.cgi) and all the siRNA sequences were synthesized by Wuhan JTS scientific (Table S3). Transfection was conducted with jetPRIME (Polyplus, France) according to manufacturer protocol.

### Fluo-3 AM experiment

Fluorescent probe loading was performed by incubating the cells with 5 µM Fluo-3 AM (Beyotime, China) for 15-60 min at 37 °C, followed by washing with PBS and then incubating in PBS for 20–30 min at 37 °C to enable complete conversion of Fluo-3 AM to Fluo-3 within the cells. The fluorescence intensity of Fluo-3 was measured with fluorescence laser plate reader (PERKINELMER, USA). The fluorescence intensity at 525 nm was used to determine the relative Ca^2+^ content (excitation 488 nm, emission 525 nm). The image of Fluo-3 intensity in the cytoplasmic calcium was captured by fluorescence microscopy.

### KB-R7943 treatment

MuSCs were incubated with 10 μM KB-R4793 for 24 h at 37 °C followed by washing with PBS and cultured in RPMI-1640 medium (Life, A10491) supplemented with 20% FBS (Gibico, #10270-106) for further analysis.

### Vector construction and overexpression experiment

The cDNA coding for isoform 1 (transcript variant 1) of *Slc8a3* was obtained from GenBank

(NM_001167920.1). The coding region of *Slc8a3* was amplified by PCR using primers (Table S2) and subcloned to the pmEGFP-C1 vector using restriction enzymes NheI and BamHI (Addgene plasmid #36412). The modified vector plasmid was confirmed by DNA sequencing and plasmid DNA was extracted by endotoxin free plasmid extraction kit (TIANGEN, China). 2 µg plasmid DNA of modified vector was diluted into 200 µL jetPRIME® buffer (Polyplus-transfection, Illkirch, France) and mixed. 4 µL jetPRIME® was added into mixed solution and incubated for 10 min at room temperature. 200 µL of transfection mix was added per well dropwise onto the cells in serum containing medium. The plates were incubated at 37 °C for 6 h and then the medium was changed.

### Whole-genome methylation-seq

Genomic DNA was extracted from isolated MuSCs and treated with EpiTect Fast Bisulfite Conversion Kits (Qiagen, 59802) according to the manufacturer’s instructions. The methylation-seq library was constructed using QIAseq Methyl Library Kit (Qiagen, 180502). The WGBS libraries were sequenced on the Illumina NovaSeq platform (Illumina, CA, USA). The sequencing adaptors and low-quality bases on read ends were trimmed by Fastq Clean reads were mapped to the mouse reference genome (mm10) using BatMeth2. DNA methylation calling was performed with BatMeth2-calmeth. BigWig files were generated for visualization using batmeth2 to bigwig. py scripts. Regions with at least 20% changes of absolute methylation level and p < 0.01 were defined as DMRs. Hypermethylated genes were obtained from DMR annotation and mm10 (UCSC, GRCm38/mm10) reference genome using BEDtools ‘intersect’ command [70].

### RNA-seq analysis

RNA-Seq data were quality checked with FastQC and low-quality sequences were removed with trimmomatic (version 0.39). We used hisat2 (version 2.1.0) to gain read alignment by mapping reads against reference genome mm10 [71]. Gene expression quantification was performed with feature Count (version 2.1.0) and gene annotation document “Mus_musculus.GRCm38.102.chr.gtf” [72]. The gene information Query and Transformation were performed by ensemble bioMart database (http://asia.ensembl.org/biomart/martview/). Differentially expressed genes (DEGs) between KO and WT samples were analysed with DESeq2 (version 1.30.1) and genes with an adjusted p-value < 0.01 and |Log_2_FC | > 1 were identified as DEGs [73]. Plots of PCA and heatmap were finished by R packages ggplot2 and heatmap. The GO (Gene Ontology) and KEGG (Kyoto encyclopedia of Genes and Genomes) functional enrichment analysis were performed by the metascape database (http://metascape.org/gp/index.html).

### Statistical analysis

Animals and tissues for all phenotypic characterization were randomly chosen among each genotype population. The cross-sectional area of TA and myofiber and the number of myofibers and the fusion index (the number of nuclei in differentiated myotubes) were counted by Image J software and Graphpad prism 6. Quantification of alizarin red staining and Oil red O were counted by Image J software For MuSCs proliferation and differentiation quantification, at least 3 microscope fields were counted for each sample. The quantification of error bars represented standard error of mean (SEM). Statistical differences between groups were determined by unpaired two-tailed *t* test in GraphPad Prism 6 software. ns indicated no significant difference, * indicated *p* < 0.05, ** indicated *p* < 0.01, *** indicated *p* < 0.001.

## Supplementary information

Supplymentary Information of Manuscript

The confirmation Email

## Data Availability

All the sequencing data (mRNA-seq, methylation-seq) in WT and Tet2 KO MuSC are available through Bioproject under the accession number PRJNA813007.
