# Supplementary material for: Disturbance of calcium homeostasis and myogenesis caused by TET2 deletion in muscle stem cells
Source: Cell Death Discov. 2022 Apr 30;8:236. doi: 10.1038/s41420-022-01041-1 (PMC9056526; doi:10.1038/s41420-022-01041-1)
Supplement: Supplementary file 1 — Supplymentary Information of Manuscript [file 41420_2022_1041_MOESM1_ESM.doc]

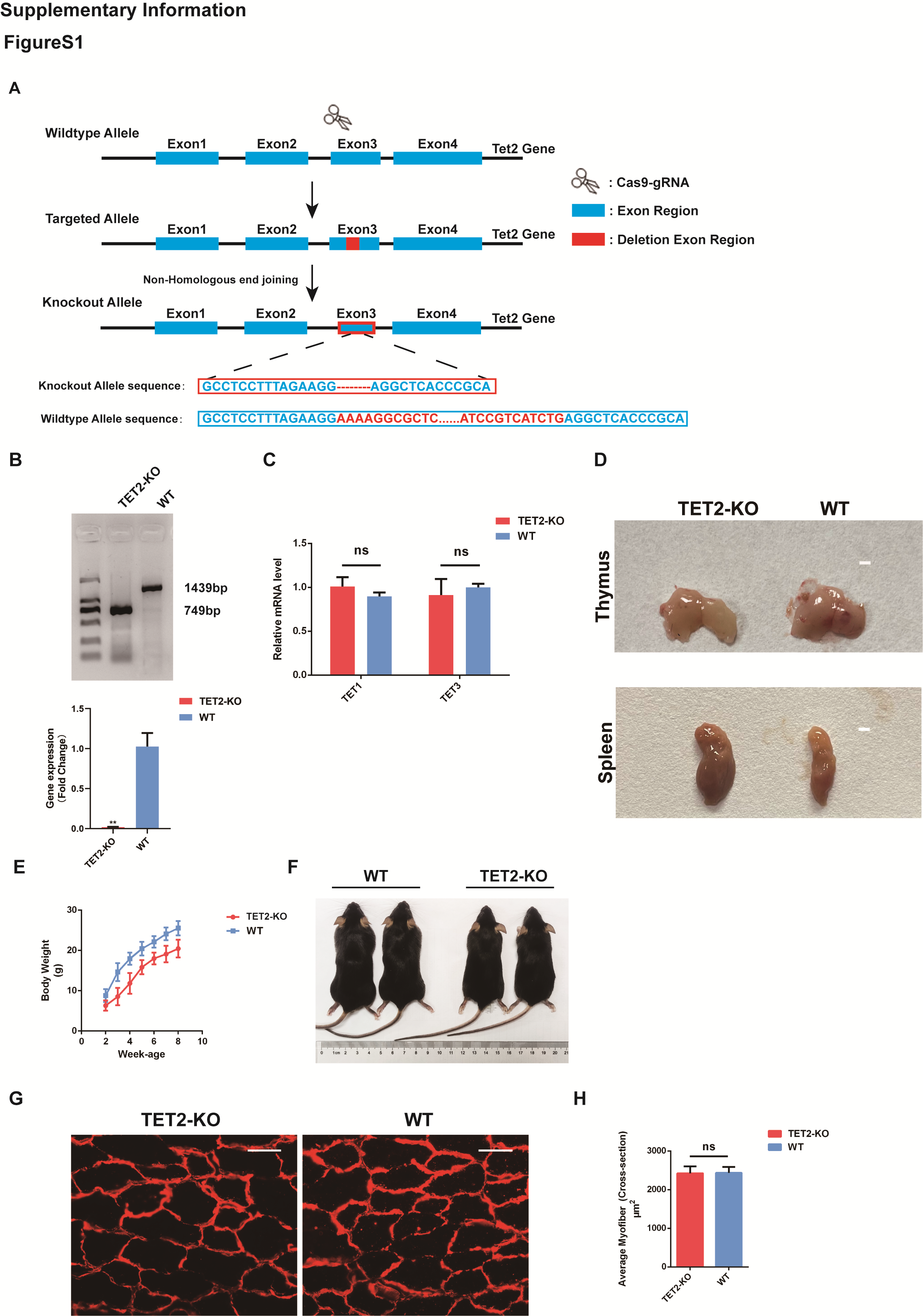


**Figure S1**

**A**: Schematic diagram of gene-editing for TET2-KO Mice**. B**: Genotyping and quantification of TET2 expression level in TET2-KO and WT mice (n = 3 biological samples). **C**: The quantification of TET1 and TET3 expression levels in muscle tissue from TET2-KO and WT mice (n = 3 biological samples). **D:** Representative images of thymus and spleen from 8-week-old WT and TET2-KO mice (Scale bars, 1 mm)**.** **E:** Body weight change profiles of 2 weeks to 8 weeks old TET2-KO and WT mice (n = 6 mice) **F:** Representative images of body size of 8 weeks old WT and TET2-KO mice. **G:** Immunofluorescence staining of Laminin of TA muscles isolated from 8-week-old WT and TET2-KO mice (Scale bars, 100 μm) **H:** Quantification of average myofiber cross-section area (n = 3 muscle samples). Error Bar indicated SEM；ns indicated that Not Significant, ** indicated that p < 0.01.

**
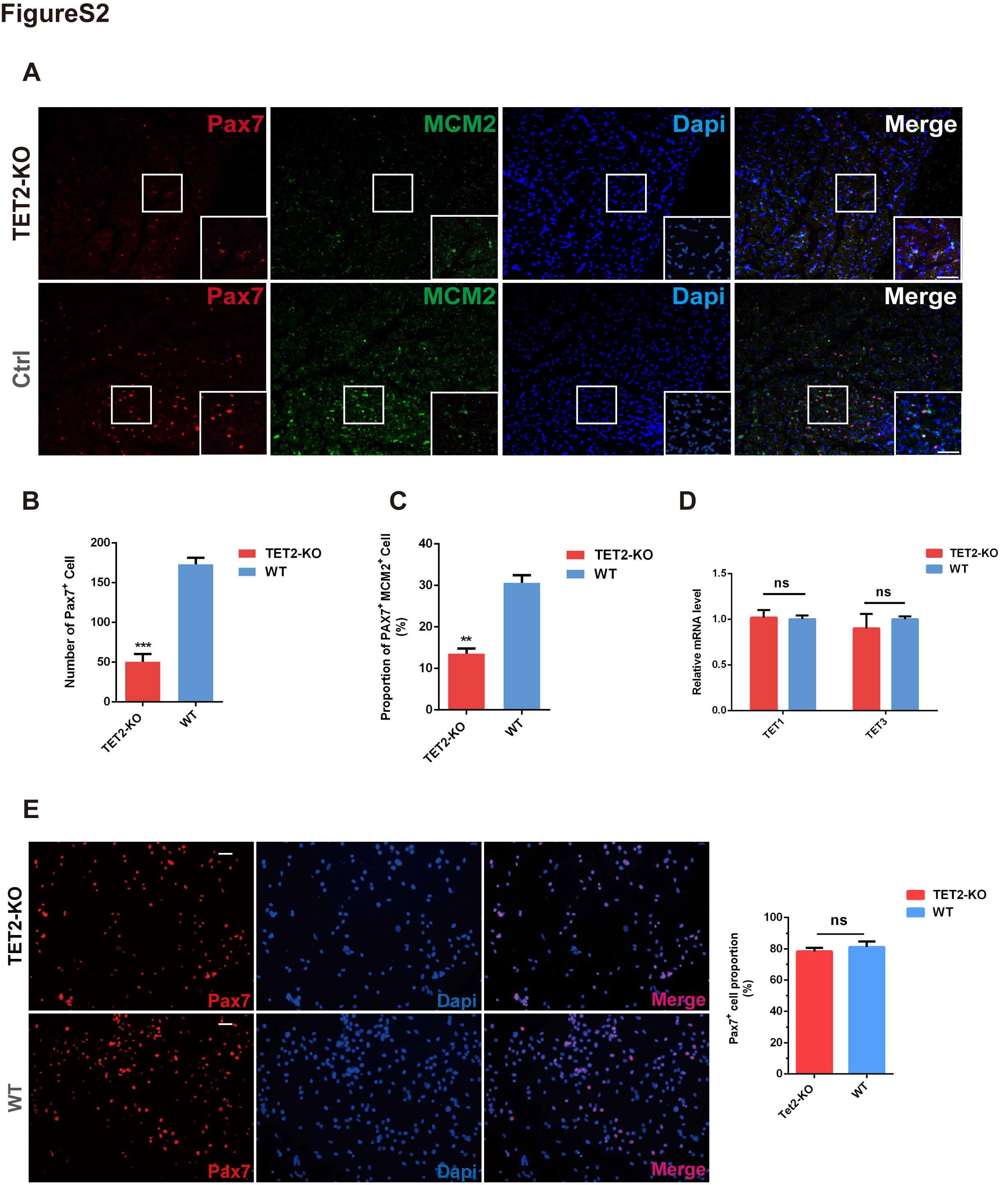
**

**Figure S2**

A: Immunofluorescence staining of Pax7 and MCM2 of TA tissues isolated from 1-week-old WT and TET2 KO mice (Scale bars, 50 μm) **B**: Quantification of the number of Pax7^+^ cells in TA muscle from 1-week-old WT and TET2 KO mice (n = 3 biological samples). **C**: Proportion of proliferative MCM2^+^/Pax7^+^ cells in TA muscle from 1-week-old WT and TET2 KO mice (n = 3 biological samples). **D**: The quantification of TET1 and TET3 expression levels in muscle stem cells (n = 3 biological samples). **E:** Immunofluorescence staining of Pax7 in fresh MuSC isolated from 8-week-old WT and TET2 KO mice (Scale bars, 50 μm) and quantification of the proportion of Pax7^+^ cells (n = 3 biological samples). Error Bar indicated SEM；ns indicated that Not Significant, ** indicated that p < 0.01, *** indicated that p < 0.001.

**
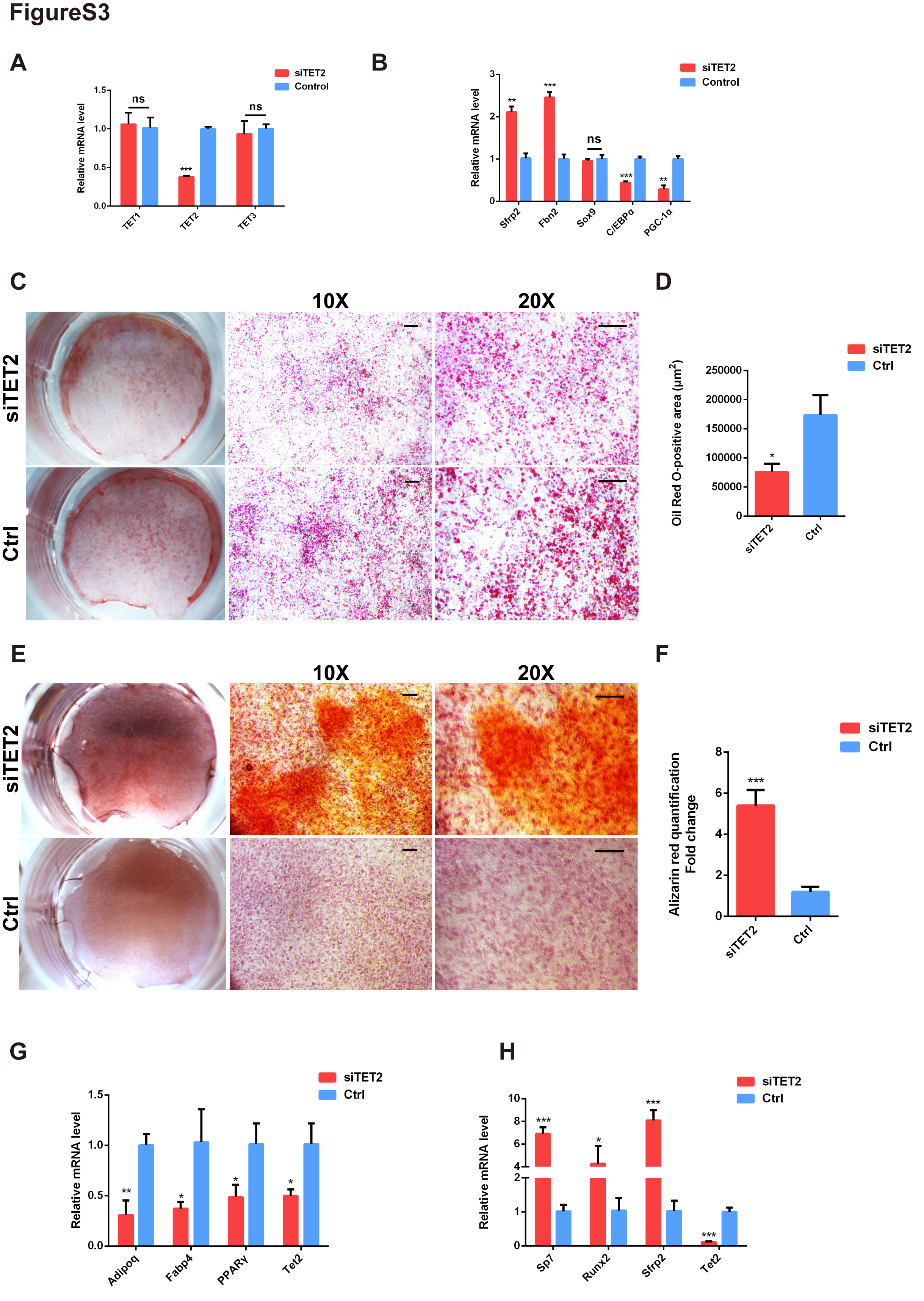
**

**Figure S3**

**A:** Relative expression levels of TET1, TET2 and TET3 in the siTET2 and control groups (n = 3 biological samples). **B:** Relative expression level of genes involved in osteogenesis (*Sfrp2, Fbn2*), chondrogenesis (*Sox9*), and adipogenesis (*C/EBPα, PGC-1α*) in the siTET2 and control groups (n = 3 biological samples). **C:** Oil red O staining of siTET2 and control groups after adipogenic differentiation (Scale bars, 100 μm). **D:** Quantification of Oil Red O positive area of siTET2 and control groups (n = 3 biological samples). **E**：Alizarin red staining of siTET2 and control groups after osteogenic differentiation (Scale bars, 100 μm). **F**: Quantification of Alizarin Red staining of siTET2 and control groups (n = 3 biological samples) **G:** Relative expression levels of adipose differentiation-related genes in siTET2 and control groups (n = 3 biological samples) **H:** Relative expression levels of osteogenic differentiation-related genes in siTET2 and control groups (n = 3 biological samples)**.** Error Bar indicated SEM. ns indicated that Not Significant, * indicated that p < 0.05, ** indicated that p < 0.01, *** indicated that p < 0.001.

**
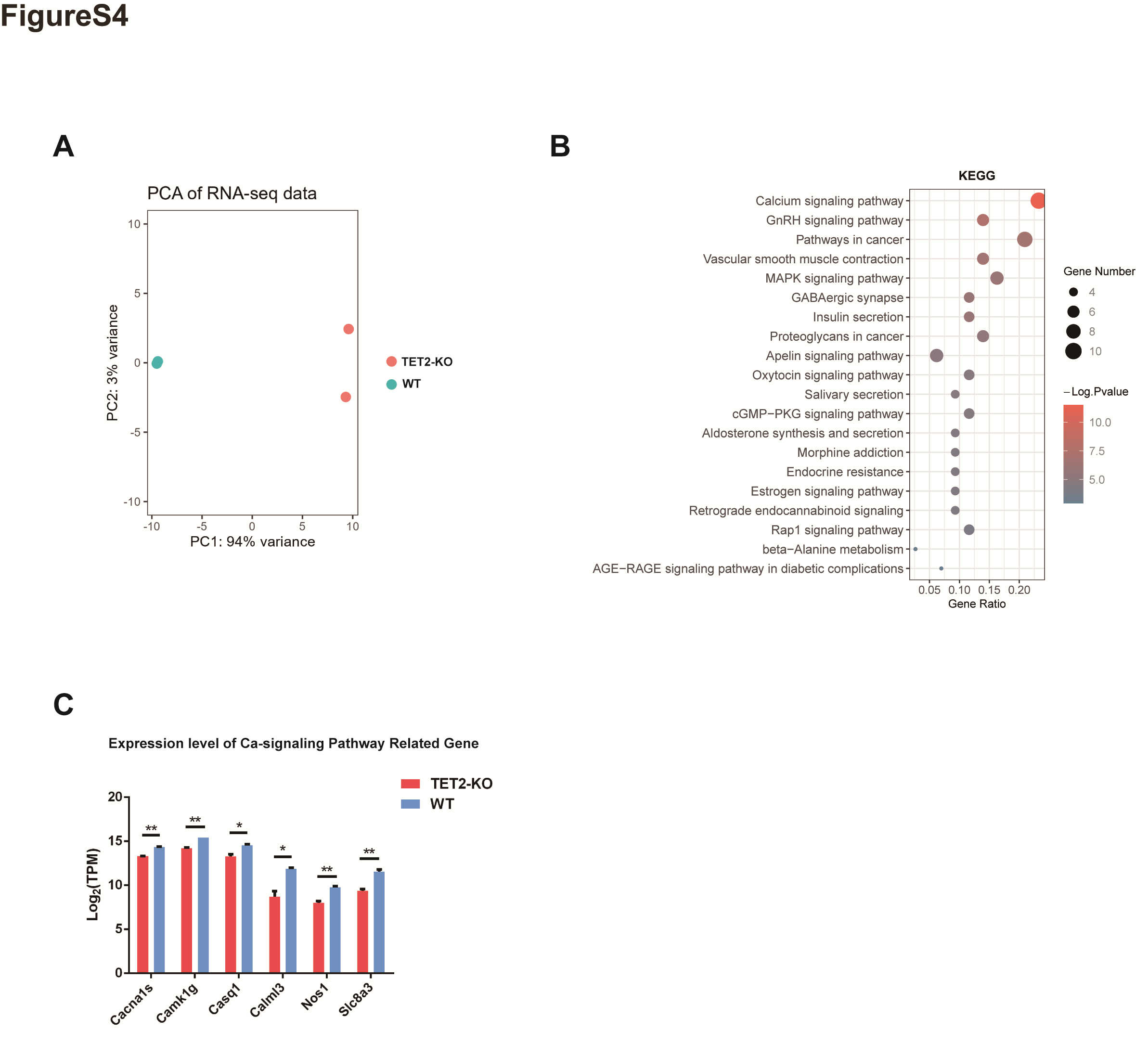
**

**Figure S4**

**A****:** PCA plots of RNA-seq data from TET2-KO and WT**. B:** KEGG analysis of downregulated gene (TET2-KO versus WT). **C:** Expression level of calcium-signaling related gene (TET2-KO versus WT, n = 2 biological samples) Error Bar indicated SEM. * indicated that p < 0.05, ** indicated that p < 0.01


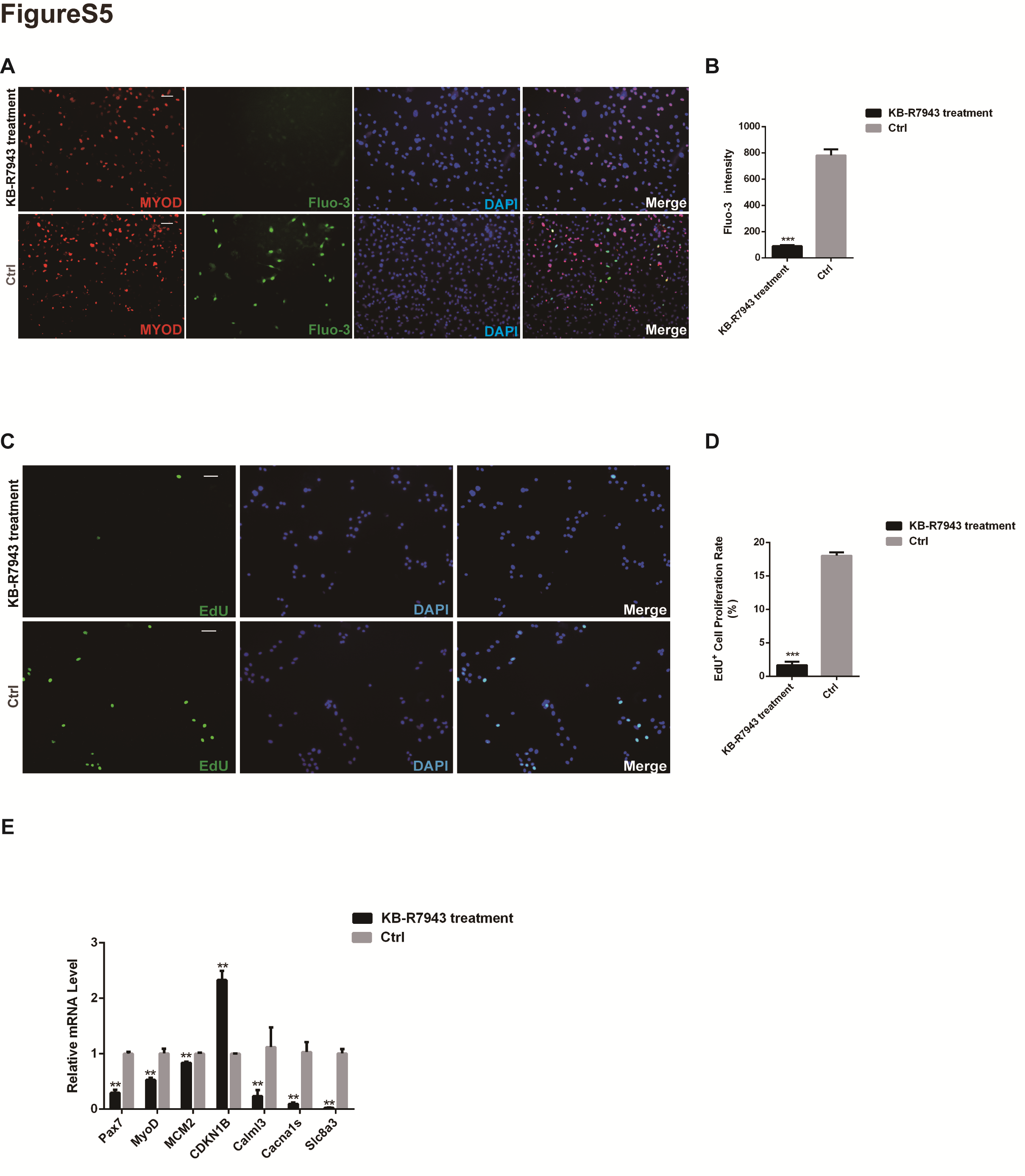


**Figure S5**

**A**: Fluorescent labeling of Fluo-3 and MyoD of WT MuSC and KB-R7943 treated MuSC. (Scale bars, 50 μm). **B**: Quantification of the intensity of Fluo-3 from WT group and treatment group (n = 3 biological samples). **C**: Fluorescence staining of EdU of WT MuSC and KB-R7943 treated MuSC. (Scale bars, 50 μm). **D**: Quantification of the percentage of EdU^+^ cell in WT MuSC and KB-R7943 treated MuSC (n = 3 biological samples). **E**: The relative expression levels of myogenic related gene (*Pax7, MyoG*), proliferative gene (*MCM2, CDKN1B*) and calcium-signaling related genes (*Calml3, Cacna1s, Slc8a3*) in WT group and treatment group (n = 3 biological samples). Error Bar indicated SEM and ** indicated that p < 0.01, *** indicated that p < 0.001.

**
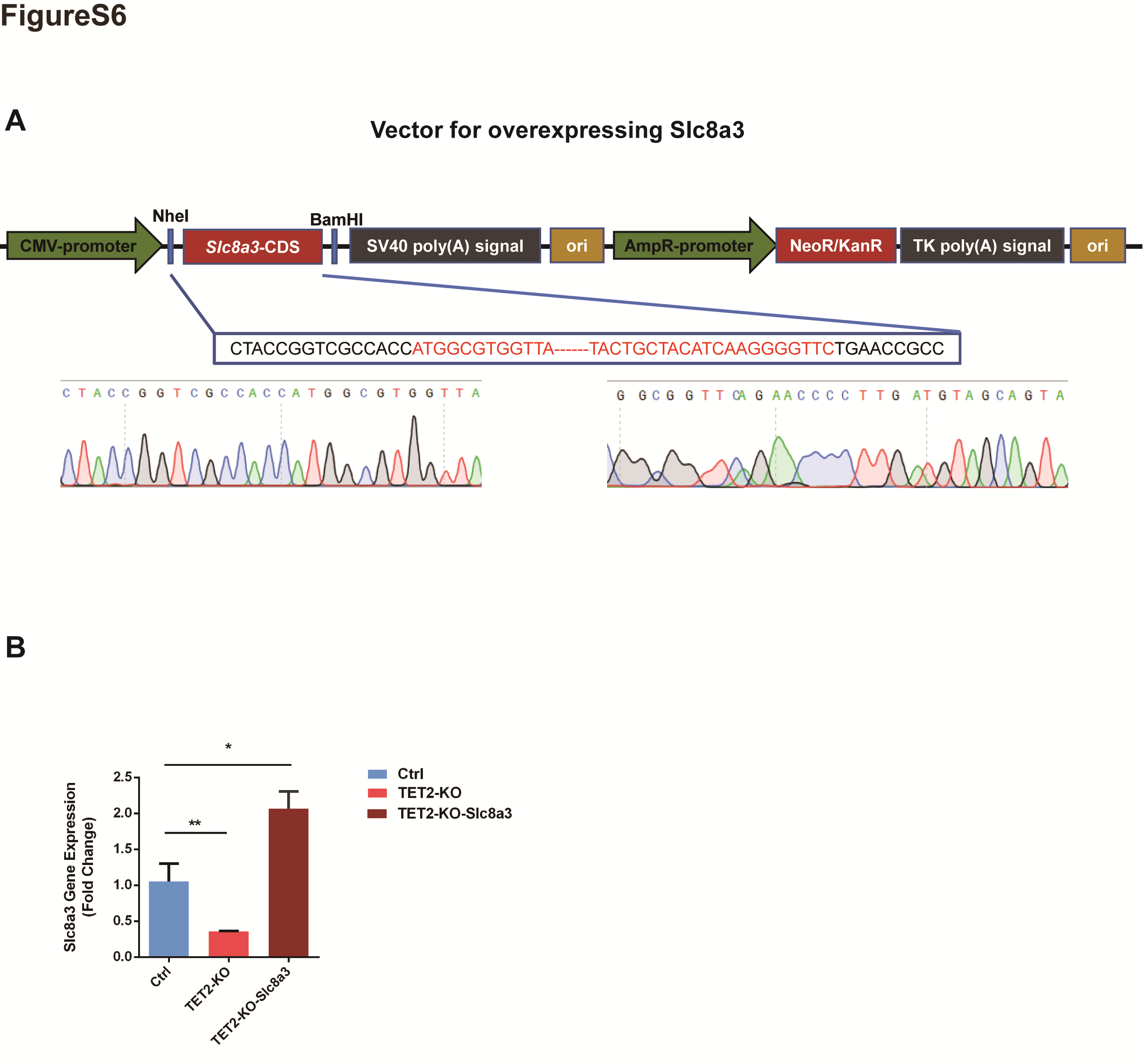
**

**Figure S6**

**A**: Construction of vector overexpressing *Slc8a3*. **B**: The relative expression levels of *Slc8a3* gene in Ctrl, TET2-KO and TET2-KO-Slc8a3 groups (n = 3 biological samples). Error Bar indicated SEM and * indicated that p < 0.05, ** indicated that p < 0.01.

**Supplementary Table**

| **TableS1：****The antibody used in this study** | | |
| --- | --- | --- |
| **Name** | **Vendor (catalogue)** | **Description** |
| PAX7 | DSHB (concentrate) | Mouse monoclonal to PAX7 |
| MyoD | Santa Cruz Biotechnology (sc-32758) | Mouse monoclonal to MyoD |
| `MHC | DSHB (concentrate) | Mouse monoclonal to MHC |
| eMHC | DSHB (concentrate) | Mouse monoclonal to eMHC |
| Laminin | Sigma (L9393) | Rabbit monoclonal to Laminin |
| MCM2 | Abcam (ab4461) | Rabbit/Polyclonal to MCM2 |
| Donkey anti-mouse IgG (H+L)  Highly cross-adsorbed secondary antibody alexa fluor 488 | Invitrogen (A-21202) | Second Antibody |
| Donkey anti-rabbit IgG (H+L)  Highly cross-adsorbed secondary antibody alexa fluor 555 | Invitrogen (A-31572) | Second Antibody |
| Goat anti-mouse IgG1  cross-adsorbed secondary antibody alexa fluor 555 | Invitrogen (A-21127) | Second Antibody |
| Donkey anti-mouse IgG (H+L) Highly Cross-Adsorbed Secondary Antibody Alexa Fluor 555 | Invitrogen (A-31570) | Second Antibody |

| **TableS2：The primers used in this study** | | | |
| --- | --- | --- | --- |
| **Applications** | **Primer name** | **Forward** | **Reverse** |
| Genotyping | TET2-KO | GTGACTAAGGCCTGTGATGCTGAT | CCCTTGGGTATTATTGTGAGTTTG |
| RT-qPCR | GAPDH | TCATCAACGGGAAGCCCATC | TCTCGTGGTTCACACCCATC |
| RT-qPCR | TET1 | TGGTGGCGTATAGCACAGTT | GGTTCAATCCCAGCACCAGA |
| RT-qPCR | TET3 | GGGCAGGCAGCGTAGC | ATGAGGTGAGCCAATGGGTG |
| RT-qPCR | TET2 | TCCCCGTCTTGTTCTTCAGC | CCTCCACTGTGGCCATGATT |
| RT-qPCR | MCM2 | ACATCGAGTCCATGATCCGC | ACGCTGAACTTCTGGGTGTC |
| RT-qPCR | Ki67 | AGCTCCTGCCTGTTTGGAAG | CTCAGCCTCACAGGCTCATC |
| RT-qPCR | CDKN1A | GCAAAGTGTGCCGTTGTCTC | CGTCTCCGTGACGAAGTCAA |
| RT-qPCR | CDKN1B | AGATACGAGTGGCAGGAGGT | ATGCCGGTCCTCAGAGTTTG |
| RT-qPCR | MyoD | GCTGGAGCTCTGAGGAGTG | TGTCCATCAGCACGAAGTCC |
| RT-qPCR | PAX7 | GTGCCCTCAGTGAGTTCGAT | CCACATCTGAGCCCTCATCC |
| RT-qPCR | MyoG | CAGCCCAGCGAGGGAATTTA | AGAAGCTCCTGAGTTTGCCC |
| RT-qPCR | Myh1 | GAAGTTGCATCCCTAAAGGCAG | CGATGACTTGGCGTCAAAAGG |
| RT-qPCR | PGC-1α | CAGCTGCCTTATTGGTTTCGTT | AGCAGCACACTGGTTGGAAG |
| RT-qPCR | C/EBPα | GCAAAGCCAAGAAGTCGGTG | TCACTGGTCAACTCCAGCAC |
| RT-qPCR | Sfrp2 | CTGCCTCCTGCATGTGTGTA | TCTGGATGGGCTTTTCGCTT |
| RT-qPCR | Sox9 | TGAAGATGACCGACGAGCAG | GGATGCACACGGGGAACTTA |
| RT-qPCR | Runx2 | TCATGGCCGGGAATGATGAG | CCTGCCTGGGATCTCGTCC |
| RT-qPCR | Fbn2 | AAATACTCCGAGGGCCCAAC | GGCGGGAACAGAATCCATCT |
| RT-qPCR | Sp7 | CTGAGAGAGGAGCAGATCCC | GTGAGCTTCTTCCTGGGTAGG |
| RT-qPCR | Adipoq | CCGCTTATGTGTATCGCTCA | ATCTTTCATGTACACCGTGA |
| RT-qPCR | Fabp4 | TTTGGTCACCATCCGGTCA | TTTCTCTTTATTGTGGTCGACT |
| RT-qPCR | Pparγ | GAAAGACAACGGACAAATCACC | GGGGGTGATATGTTTGAACTTG |
| RT-qPCR | Calml3 | CGCGAAGGGATCCGTAGGAA | ACGGTGCCGTTTCCATCTTT |
| RT-qPCR | Slc8a3 | GGTGGCGAGGACTTTGAAGA | CACTTCTGATATTCCACGTTCCA |
| RT-qPCR | Cacna1s | GAGAGAGTAATCCTCCCGCC | ACAGGTTTCTTCGGCTGCTT |
| Amplification | Slc8a3_CDS | CCAGAACCTAAGTCTTGTGT | TCTTGTTCCGGCGGTTCAGAA |

| **Table S3 siRNA sequence in this study** | |
| --- | --- |
| **Name** | **Sequence** |
| si-TET2-1 | GCAGCUCAACAGAGGUAUUTTAAUACCUCUGUUGAGCUGCTT |
| si-TET2-2 | GCACCAGACUGAGUCCAUUTTAAUGGACUCAGUCUGGUGCTT |
| si-TET2-3 | CCCUCUAAGUACUUAAGUUTTAACUUAAGUACUUAGAGGGTT |
| si-Control | UUCUCCGAACGUGUCACGUTTACGUGACACGUUCGGAGAATT |
