# Supplementary material for: Disturbance of calcium homeostasis and myogenesis caused by TET2 deletion in muscle stem cells
Source: Cell Death Discov. 2022 Apr 30;8:236. doi: 10.1038/s41420-022-01041-1 (PMC9056526; doi:10.1038/s41420-022-01041-1)
Supplement: Supplementary file 2 — The confirmation Email [file 41420_2022_1041_MOESM2_ESM.docx]

Dear Editor

I am Haoyuan Zhang, I agree with the changes to the manuscript and I have confirmed to other authors of this article by email that they have no objection to the manuscript changes.

Yours sincerely

Haoyuan Zhang

Following are the confirmation emails

**The confirmation from Heng Wang**

**The confirmation from Sheng Wang**

**The confirmation from Qiangwei Zhou**

**The confirmation from Yinlong Liao**

**The confirmation from Wenzhe Luo**

**The confirmation from Zhelun Peng**

**The confirmation from Ruimin Ren**
